# Real-Time Measurement of Ocular Wavefront Aberrations in Symptomatic Subjects

**DOI:** 10.1155/2018/9415751

**Published:** 2018-03-29

**Authors:** Sandra Franco, Jessica Gomes

**Affiliations:** Centre of Physics, University of Minho, Campus de Gualtar, 4710-057 Braga, Portugal

## Abstract

The purpose of this work was to study the real-time changes of the optical properties of the eye with accommodation in subjects with symptoms of accommodative disorders. From ocular aberrations, it is possible to compute several parameters like the response and lag of accommodation. The ocular aberrations were measured in 4 subjects, with different accommodative disorders, during several cycles of accommodation/disaccommodation and for different accommodative stimuli. The measurement was done continuously and in real time during different accommodative stimuli. It was possible to see the changes in accommodative response during the several stimuli of accommodation. Subjects with accommodative disorders showed different accommodative responses. The use of wavefront ocular aberrations can be a tool to diagnose accommodative disorders. In some subjects with complaints, this method showed irregularities even when the results of the usual clinical exams were normal.

## 1. Introduction

Aberrometry is one of the techniques available for the measurement of the optical quality of the eye. It has been clinically applied and shown to be an important diagnosis and evaluation tool. The eye aberrations are affected by several factors, such as age, pupil diameter, refractive error, and the lens accommodation. To have better knowledge of the human eye, it is important to analyze these changes in the ocular aberrations.

Aberrometry uses a wavefront sensor and the results are expressed in Zernike polynomials. The aberrations are susceptible to several factors, such as age, pupil diameter, refractive error, and accommodation, and it is important to analyze these variations for better knowledge of the human eye [1–12].

Accommodation is the process by which the lens changes its power, allowing the eye to focus objects at different distances. Several studies found changes in retinal image quality when accommodation was stimulated and although there are some variations between individuals, these studies show that monochromatic aberrations increase with accommodations levels [7, 13–15]. The spherical aberrations have been reported to change in the negative direction and high-order ocular aberrations have been reported to increase with the accommodation level [8, 16, 17]. This variation can be explained due to changes in the crystalline lens shape during accommodation [15, 18].

Through aberrometry, it is possible to calculate objectively accommodative parameters, such as the accommodative response or accommodative lag [19, 20]. Recently, some authors have evaluated the dynamic properties of accommodation using wavefront sensors [21, 22].

Accommodative dysfunctions are a common visual problem, even in populations of university students [23–28]. It was estimated that 10.8% of the university students had an accommodative excess and in 6.2% the accommodative insufficiency was present [27].

In this paper, we present new methodology to assess the ocular aberrations' changes with the accommodation eye. This methodology allows* in vivo* and real-time measurement of ocular aberrations' changes for different accommodative step stimuli. There are also presented results obtained from real observers, focusing on the applications of this technique to symptomatic patients who do not present changes or findings in the regular optometric examination but that are detected by applying this methodology.

## 2. Materials and Methods

### 2.1. Aberrometer Setup

To measure the ocular wavefront aberrations in real time with different accommodation demands, an in-house Hartmann-Shack aberrometer (Thorlabs WF150-7AR) with a resolution of 1280 × 1024 and 39 × 31 lenslets working at frequency of 15 Hz was used. The optical beam was generated by a super luminescent diode (SLD) with a spectral maximum at 830 nm. The power of the SLD radiation at the eye was 12 *μ*W. The beam diameter in the wavefront sensor was around 4 mm and the effective diameter used for measuring the aberrations was 2 mm.

### 2.2. System for Varying the Accommodation

**Figure 1:**
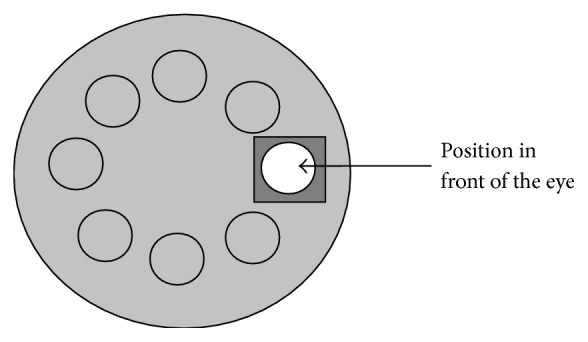
Motorized system adapted to the Hartmann-Shack aberrometer.

A motorized system (MS) that contains 8 positions (positions 0 to 7), where lenses with different powers were placed (Figure 1 and Table 1), was adapted to the aberrometer. The MS rotates, placing the different lenses in front of the subject eye, stimulating the accommodation.

The lenses' powers used were selected taking into account the amplitude of accommodation of all the subjects, which was previously obtained in an optometric examination. Lenses were placed 20 mm in front of the subject eye.

The position of the MS was synchronized with the ocular wavefront aberration measurement and controlled by the same software.

### 2.3. Participants

Four subjects (3 females and 1 male) were recruited from the staff and students of the Physics Department of University of Minho. Their ages ranged from 22 to 45 years (29 ± 10.9 years). None of the subjects presented history of ocular pathology and had never undergone either ocular surgery or orthokeratology.

The study adhered to the tenets of the Declaration of Helsinki and was approved by the Ethical Subcommission of Life and Health Science of University of Minho. After the explanation of the procedures, an informed consent was obtained from all the subjects.

Initially, all the participants underwent a visual optometric assessment. The exam contained ocular health evaluation, monocular and binocular distance visual acuities, and refractive examination to determine their ocular refractive state. Amplitude of accommodation (by the Sheard method), monocular estimated method (MEM) retinoscopy, and near monocular accommodative facility with ±2.00 D flippers were also evaluated.

### 2.4. Data Collection

**Figure 2:**
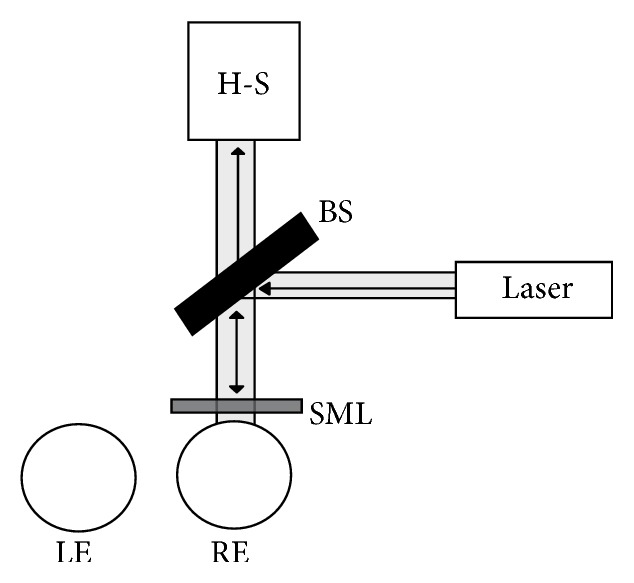
Schematic representation of the experimental procedure. H-S is the Hartmann-Shack wavefront sensor, BS is the beam splitter, LE is the left eye, and RE is the right eye.

Subject's head was stabilized on the chin rest and the right eye aligned with the system. The subject was asked to fixate a small point in front of the eye and to keep it focused. The fixation target was a small red light spot with a diameter of 1 mm. The ocular aberrations were measured in the same eye through which the accommodation was stimulated (Figure 2), while the other eye was occluded. The first position of the MS placed in front of the eye was position 0 and the last was the 7th, realizing thus a cycle. For subject D, it was done in reverse order, that is, from position 7 to position 0. Each position was kept for nearly 5 seconds. Measurements were performed in natural pupil (without the use of cycloplegic) and in mesopic conditions. During the procedure, the subjects were instructed to blink to provide a stable lacrimal film.

### 2.5. Data Analysis

After each measurement, the ocular aberration values were exported by means of Zernike coefficients up to the 6th order. All the studied parameters were then computed from these data.

The fixation point was considered at infinity, so the corresponding object's vergence *L* at the lens is

(1) $$\begin{array}{c} L=\frac{1}{\infty }=0\;D. \end{array}$$

Refracting at the lens gives *L*′:

(2) $$\begin{array}{c} L^{\prime }=L+F=F, \end{array}$$

where *F* is the power of the lens.

Considering the distance from the lens to the eye as 0.020 m, the accommodative stimulus (AS) is given by

(3) $$\begin{array}{c} AS=\frac{F}{1-0.02F}. \end{array}$$

After obtaining the accommodative stimulus for each lens, the subjects' accommodative responses were then computed for each stimulus.

The accommodative (Ac) response was calculated by means of the least square fitting method, using only the Zernike defocus (*Z*_0_^2^) coefficient (see (4)) and with defocus (*Z*_0_^2^) and spherical aberration (*Z*_0_^4^) coefficients (see (5)) [29]:

(4) Ac=−4√3×Z20r2−ER,

(5) Ac2=−Z204√3+Z4012√5r2−ER,

where ER is the relaxed eye refraction, *r* is the pupil radius, and *Z*_2_^0^ and *Z*_4_^0^ are the defocusing and spherical aberration of the participant, respectively. To compute the subjects' defocus, it was necessary to calculate first the defocusing of each lens and then deduct it from the defocusing obtained during the measurements. The measured defocusing includes the ocular defocusing and the lens defocusing.

The accommodation lag (AL) is given by

(6) $$\begin{array}{c} AL=AS-AR. \end{array}$$

A positive AL corresponds to an accommodation lag and negative AL to an accommodative lead.

Root mean square of lower-order aberration (RMS LOA) and Root mean square of higher-order aberration (RMS HOA) were also analyzed.

The time to achieve an accommodative response was also computed. To this end, an exponential function (see (7)) was fitted to an accommodative trace for each specific accommodative demand [30]:

(7) $$\begin{array}{c} y=y_{0}+a\left(\;1-e^{t/\tau }\;\right), \end{array}$$

where *y* represents the accommodation response, *y*_0_ is the initial value of accommodation, *a* represents the amplitude of the accommodative response, *t* is time in seconds, and *τ* represents time constant.

The exponential functions were fitted to the data using Mathematica® software (Wolfram Research Inc.), version 11.1.1.0.

For statistical analysis, the program R version 3.3.2 was used and a confidence interval of the 95% and statistical significance when *p* value ≤ 0.05 were considered.

## 3. Results

The new methodology was tested in 4 participants (3 females and 1 male) with a mean (±SD) age of 28 (±2) years (range from 22 to 45 years).

The results of the preliminary visual examination performed to all the participants are presented in Table 2.

**Figure 3:**
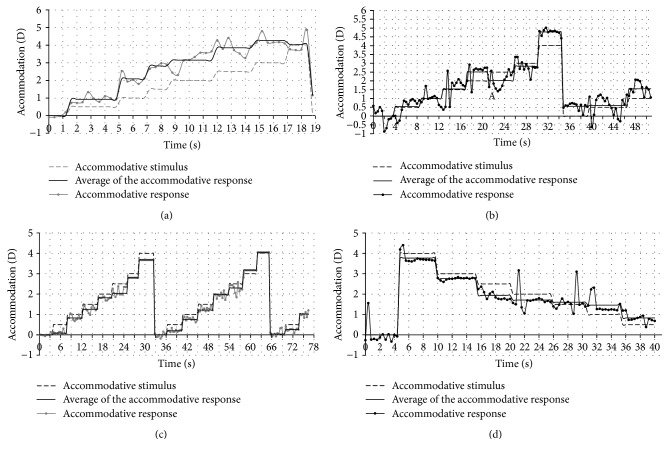
Accommodative response of all the participants over time for several accommodative stimuli and its average for each accommodative demand.

Figure 3 shows the accommodative response overtime for all the participants and for the different accommodative stimuli and their averages for each accommodative demand.

For subjects A and B (Figures 3(a) and 3(b)), the accommodative response was always higher than its stimulus. When returning to the lens of 0 D (for the second cycle), the accommodative response was higher than the initial response: on average, with no accommodative stimulus (0 D) in the first cycle, the accommodative response was 0 D, and in the second cycle, the accommodative response was 1.22 D and 0.525 D, respectively, which means that the accommodation was not totally relaxed.

The accommodative response of subject C for all cycles is shown in Figure 3(c). The accommodative response was 0 D in the first cycle with no accommodative demand (0 D) and slightly lower than the stimulus in the remaining lenses. When the lens of 0 D is again placed in front of the eye, contrary to subjects A and B, subject C can relax the accommodation and get a response of approximately 0 D.

Figure 3(d) shows the accommodative response of subject D over time and for the different accommodative stimuli. In the lenses of 0 D, the accommodative response was approximately 0 D and when placing the lenses of −4 D, the subject did not reach this accommodative demand and his response was lower. For the lenses from −3.00 D to −2.00 D, the accommodative response was also less than the stimulus. Thenceforth, accommodative response remained somewhat constant and was above the stimulus in lenses −1.50 D, −1.00 D, and −0.50 D.

**Figure 4:**
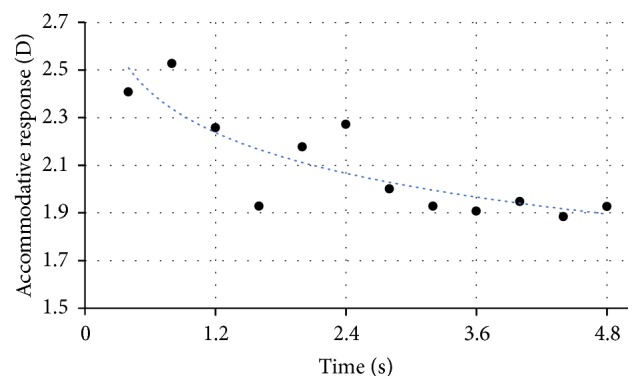
Accommodative response of subject D during time for an accommodative stimulus of 2,50 D.

Through Figure 4, it is possible to observe that subject D, in the higher accommodative demands (like 2.50 D), can, initially, obtain a response equal to the stimulus, but this is not maintained for a long time and the response decreases for lower values.

**Figure 5:**
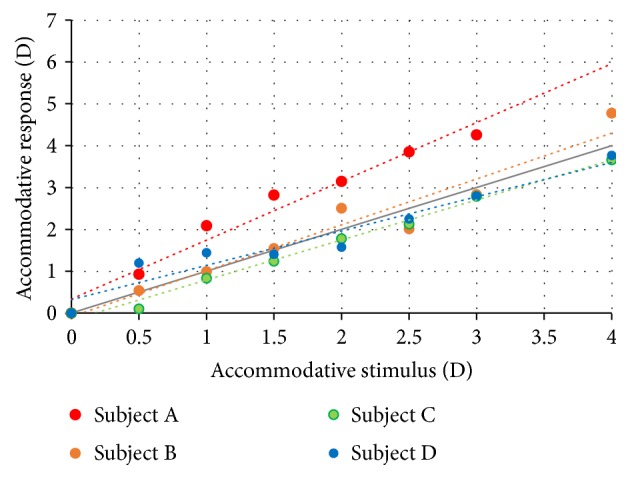
Average of accommodative response for each of the 8 accommodative stimuli for all the participants.

Figure 5 represents the averages for the accommodative responses for each accommodative stimulus and for all the 4 participants. The linear correlation variates from *r* = 0.97 for subject D to *r* = 0.99 for subject C. The grey line represents an ideal condition, where the response is equal to the stimulus.

**Figure 6:**
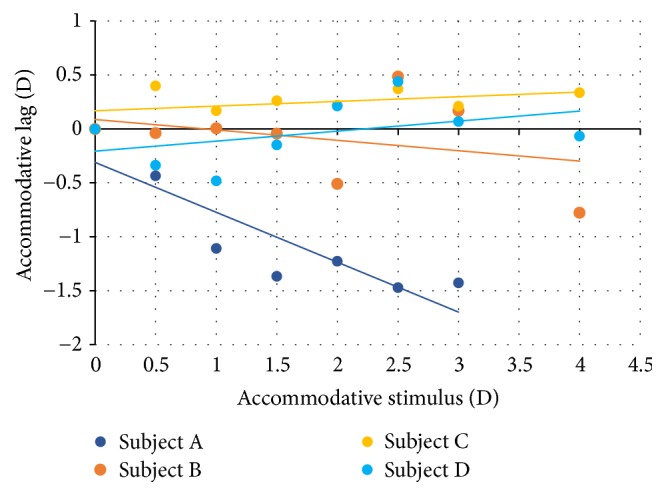
Accommodative lag as a function of accommodative stimulus. The positive values are the lag of accommodation and negative values are the lead of accommodation.

The accommodative lag was also computed and is shown in Figure 6. Subject A presents an accommodation lead that increases up to the lens of −1.50 D and, thenceforth, remains constant (approximately −1.25 D) up to the lens of −4.00 D, where it decreases to a small accommodative lag (0.18 D).

The lag of accommodation of subject C was approximately constant for all the accommodative stimuli (the slope was 0.043). The mean value of the accommodative lag was +0.22 D, next to the value found in the MEM retinoscopy (+0.50 D).

Subject D, with presbyopia, presented an increasing accommodative lag for the higher stimulus. However, for the lower stimulus, this subject presented a small lead of accommodation. In this participant, measurements were done in reverse order, that is, from the higher stimulus to OD. During the higher accommodative stimulus, the participant made a great effort to accommodate and was not able to fully relax the accommodation when a lower accommodative stimulus was presented.

**Figure 7:**
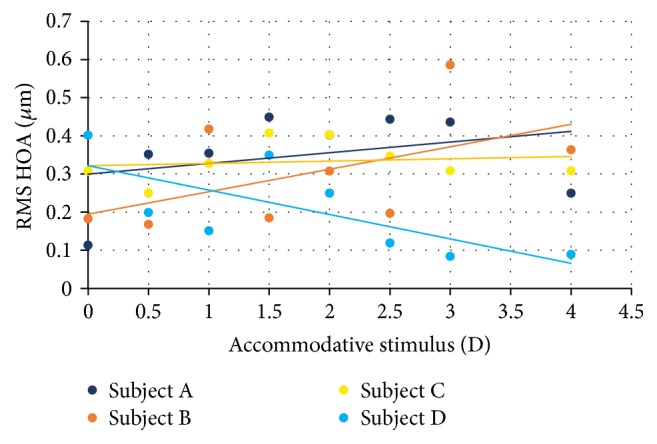
RMS of high-order aberrations (HOA) as a function of accommodative response for all the participants.

The RMS for the high-order aberrations increases significantly when the accommodation is stimulated, comparatively to the relaxed state (Figure 7). For subject A, the linear correlation (*r* = 0.7 and *p* = 0.02) was statically significant, with a slope of 0.054. In subject B, there is an increase in the RMS for high-order aberrations (*r* = 0.50 and 0.05 slope, *p* > 0,05); however, it was not statistically significant. Subject C also showed the same trend with linear correlation of 0.173 and slope of 0.007 (*p* > 0.05). Contrary to other subjects, subject D showed a decrease of the RMS of high-order aberrations with the accommodative response, with a linear correlation of −0.828 and slope of −0.087 (*p* = 0.03).

**Figure 8:**
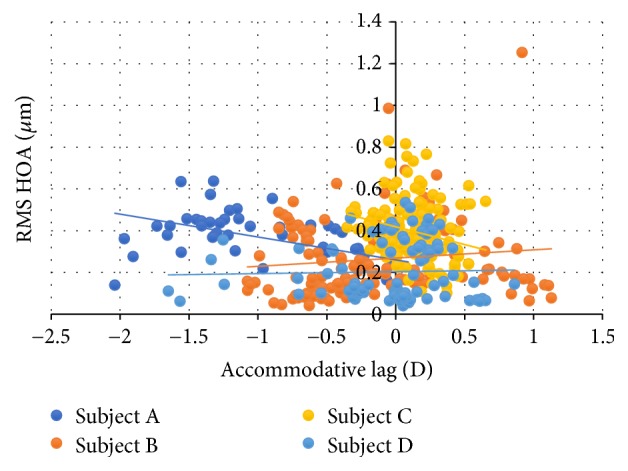
RMS of higher-order aberrations (HOA) as a function of the accommodative lag.

The RMS variation of higher-order aberration with the lag of accommodation (Figure 8) was not statistically significant for any subject.

**Figure 9:**
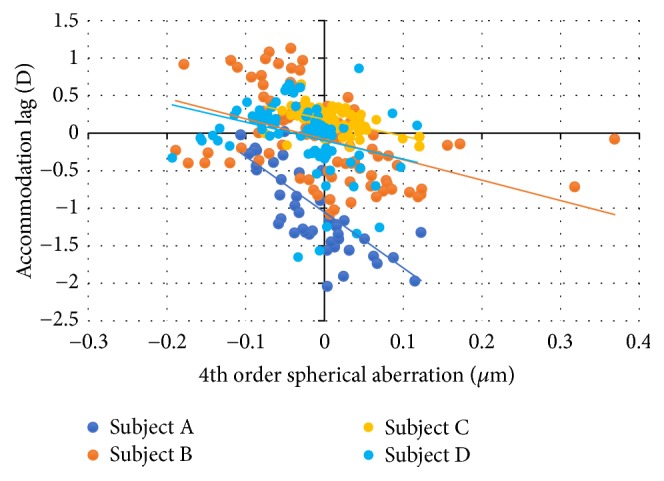
Accommodative lag as a function of 4th-order spherical aberration for all the participants.

Correlation between the accommodative lag and 4th-order spherical aberration for all subjects was found. The 4th-order spherical aberration showed more positive values for higher accommodative leads (Figure 9). The correlation was −0.607, −0.435, −0.622, and −0.26, and the slope was −7.416, −2.721, −2.230, and −2.461 in subjects A, B, C, and D, respectively.

Like subject A, the 4th-order spherical aberration had more positive values when the accommodative lag was negative, that is, when there is lead of accommodation. The linear correlation (*r*) was −0.435 and the slope was −2.721.

For subject C, the 4th-order spherical aberration remains constant with the accommodative lag, with −0.033 of linear correlation and −0.096 of slope. For subject D, like the other subjects, there is a trend for positive values of 4th-order spherical aberration with lead of accommodation, with a linear correlation of −0.26 and slope of −2.294 (Figure 9).

**Figure 10:**
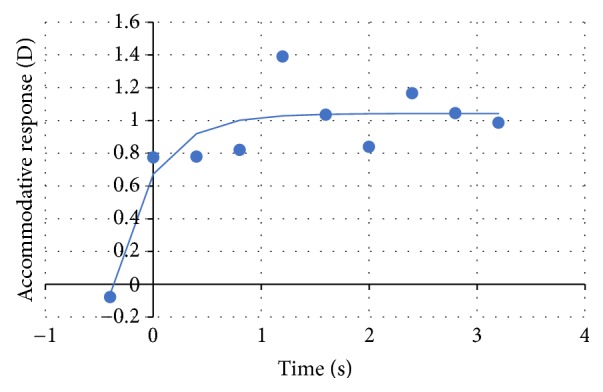
Accommodative response overtime for the lens of −0.50 D. The blue line represents the exponential equation (see (8)) fitted to the data (blue points).

The results obtained by this method also allow the measurement of the time taken to achieve a stable accommodative response. In Figure 10, the accommodative response is presented for an accommodative stimulus of 0,50 D overtime. The blue line represents the exponential equation (see (8)) fitted to the data (blue points):

(8) $$\begin{array}{c} y=0.673+0.370\left(\;1-e^{-2.73x}\;\right). \end{array}$$

In this case, subject A took 0.79 s to achieve a stable accommodation response. After the accommodative response of 1.04 D is achieved, it is possible to see that it fluctuates around this value. These fluctuations have amplitude of 0.40 D and a frequency up to 0.86 Hz.

## 4. Discussion

To variate the accommodative demand, we create a system, called VML, with different negative lenses, ranging from −0.50 D to −4 D and a neutral lens (0 D). This system was placed in front of the right eye and stimulated the accommodation. A Shak-Hartmann wavefront sensor was used to measure ocular aberrations. Subsequently, accommodative parameters were obtained. Previous studies [17, 31, 32] have reported possible direct or indirect applications of ocular aberrations' measurements; however, they do not relate specific cases. In this study, it was possible to describe the application of ocular aberrations' measurements to real cases.

Subject A has symptoms of blurred vision in far conditions after some time performing a near-vision task. Although all the previous clinical exams' results have demonstrated normal values for age, amplitude of accommodation, MEM, and accommodative facility, by these exams, it was not possible to detect any accommodative dysfunction. When the accommodative response was observed for these subjects, it was found that both subjects A and B presented a higher accommodative response than the stimulus for all the accommodative stimuli.

If we observe the accommodative response for each stimulus, it is possible to confirm that accommodative response is not instant; subject A took 1.61 s to achieve a stable accommodative response.

Subject B also showed the accommodative response greater than the stimulus on all lenses of experience 2 and almost all on experience 1 (except on the lenses of −2.50 and −3.00 D). In this subject, each lens was maintained in front of the eye for more time and so the accommodative response for each stimulus was better observed. It was also possible to observe the fluctuations of accommodation, which were higher in higher accommodative demands, supporting the previous study [33].

In both subjects, after the accommodation was stimulated, they were unable to relax the eye, and when the lens of 0 D was again placed, the accommodative response was greater than 0 D and greater than the initial response (in the first cycle) for this same lens. If we observe the plots of accommodative lag as a function of accommodative response for these two subjects, A and B, we can observe that the higher accommodative responses correspond to higher stimuli, that is, to higher accommodative lead. Subject C, which did not have symptomatology and presented normal values in the optometric evaluation, had accommodative response lower than the stimulus, similar to the information founded in the MEM exam. Furthermore, after stimulating the accommodation in 4 D, he was able to completely relax it when the 0 D lens was placed in front of the eye. This did not occur with the subjects with symptoms.

Subject D, being a presbyter and therefore with lower amplitude of accommodation, had a response lesser than the stimulus in the more accommodative demands (lenses of −2 to −4 D). In these lenses, the response was, initially, higher but this response is not maintained and soon presents lower responses. This method allows also calculating for how long the response is maintained.

The two ways of calculating the accommodative response, with or without spherical aberration, did not show statistically significant differences in general. However, when the spherical aberration is used, the response tends to be higher.

The RMS of high-order aberrations was higher when the accommodation was stimulated, observed in subjects A, B, and C. These results agree with several previous studies [7, 17, 34]. In subject D, the opposite is observed: when the accommodation was stimulated, the RMS of high-order aberrations was lower.

Both measures on subject B did not show significance between them, in low- and high-order aberrations, and obtained the same behaviour of the accommodative response, that is, the values of the response, in general, higher than the stimulus and the inability to relax accommodation when the 0 D lens is again placed after being stimulated until 4 D. This fact showed a good precision of the methodology. Subjects A and B, who presented similar symptomatology and results in the optometric exam, presented identical variations of ocular aberrations, accommodative response, and accommodative lag.

The 4th-order spherical aberration tends to have positive values with lead of accommodation, observed in all subjects, except for subject C. This fact supports the previous studies of Plainis et al. [33].

The RMS of the total high-order aberrations showed no statistically significant changes with the lag of accommodation in all of the subjects.

With the data obtained, it was possible to measure the accommodative response time, as well as the frequency and the amplitude values for the accommodation microfluctuations. An example was given and the results obtained for subject A were in accordance with the values presented by other researchers [35, 36].

It is also important to understand an important limitation of the technique: the use of the red stimulus could have caused greater accommodative stimulus and, consequently, higher accommodative responses. Due to the chromatic aberrations of the human eye, there is a higher accommodation when the subject reads with red light [37].

## 5. Conclusions

In this work, a methodology was presented to vary the accommodation and obtain the aberrations up to the 6th order in real time. Through the ocular aberrations, it was possible to calculate accommodative parameters, such as accommodative response, lag, and lead of accommodation and better study of behaviour of the accommodation in different situations, such as in symptomatic patients, but without anomalies in clinical exams usually performed in optometric practice and in prepresbyopic patients and patients without symptomatology or anomalies in clinical exams.

Patients with complaints of blurred vision at far after performing a near-vision task showed an accommodative response higher than the stimulus. These accommodative leads were greater for higher demands. These subjects also had difficulties in totally relaxing the accommodation after making an accommodative effort which is in agreement with the complaint of having difficulty seeing in the distance after performing a task in near-vision. This does not occur in the patients with normal optometric exams and without symptoms. We suggest that a large part of this problem is not detectable in clinical optometric practice and, for this, can receive wrong diagnosis and consequently a less appropriate treatment. This method allowed detecting anomalies in the ocular accommodation that the clinical examination did not present. The measurement of wavefront ocular aberrations can be a tool to diagnose accommodative disorders.

The RMS of high-order aberrations showed greater values when the accommodation is stimulated in no presbyopic patients.

Calculating the accommodative response with 4th-order spherical aberration results in a little greater values, but, in general, there are no statistically significant differences.

Another application of this technique is the possibility to measure the accommodative response time as well as to analyze its fluctuation during a period of time. In addition, it may be useful in evaluating the results of visual therapy programs in the treatment of this type of dysfunction.

## Figures and Tables

**Table 1:** Power of the lenses and accommodative stimulus for each position of the MS.

Motorized system		
Position	Power of the lens	Induced accommodation from the lens
0	0 D	0 D
1	−0.50 D	0.50 D
2	−1.00 D	0.98 D
3	−1.50 D	1.46 D
4	−2.00 D	1.92 D
5	−2.50 D	2.38 D
6	−3.00 D	2.83 D
7	−4.00 D	3.70 D

**Table 2:** Results of the visual examination for all the participants.

	Age	RE (D)	Am (D)	MEM retinoscopy (D)	AF (CPM)	Observations
Subject A	22	+0.50	9.00	+0.50	19	Far blurred vision after performing a near-vision task

Subject B	22	+0.50	9.00	+0.50	19	Far blurred vision after performing a near-vision task

Subject C	28	+0.50	8.50	+0.50	12	No symptoms

Subject D	45	0.00	4.00	+1.00	1	Difficulty focusing on near objects. Far blurred vision after performing a near-vision task.

Am: amplitude of accommodation; RE: spherical equivalent refractive error; AF: accommodative facility; CPM: cycles per minute; MEM retinoscopy: monocular estimated method retinoscopy.
